# The Role of Carbonic Anhydrase αCA4 in Photosynthetic Reactions in *Arabidopsis thaliana* Studied, Using the Cas9 and T-DNA Induced Mutations in Its Gene

**DOI:** 10.3390/plants11233303

**Published:** 2022-11-29

**Authors:** Natalia N. Rudenko, Natalya V. Permyakova, Lyudmila K. Ignatova, Elena M. Nadeeva, Alla A. Zagorskaya, Elena V. Deineko, Boris N. Ivanov

**Affiliations:** 1 Institute of Basic Biological Problems, Federal Research Center «Pushchino Scientific Center for Biological Research of the Russian Academy of Sciences» Pushchino, Moscow Region 142290, Russia; 2 Institute of Cytology and Genetics, Siberian Branch of Russian Academy of Sciences, Novosibirsk 630090, Russia

**Keywords:** carbonic anhydrase, photosynthesis, *Arabidopsis thaliana*, gene expression, thylakoids, CRISPR/Cas9, T-DNA-mutants

## Abstract

An homozygous mutant line of *Arabidopsis thaliana* with a knocked out *At4g20990* gene encoding thylakoid carbonic anhydrase αCA4 was created using a CRISPR/Cas9 genome editing system. The effects of the mutation were compared with those in two mutant lines obtained by the T-DNA insertion method. In αCA4 knockouts of all three lines, non-photochemical quenching of chlorophyll *a* fluorescence was lower than in the wild type (WT) plants due to a decrease in its energy-dependent component. The αCA4 knockout also affected the level of expression of the genes encoding all proteins of the PSII light harvesting antennae, the genes encoding cytoplasmic and thylakoid CAs and the genes induced by plant immune signals. The production level of starch synthesis during the light period, as well as the level of its utilization during the darkness, were significantly higher in these mutants than in WT plants. These data confirm that the previously observed differences between insertional mutants and WT plants were not the result of the negative effects of T-DNA insertion transgenesis but the results of *αCA4* gene knockout. Overall, the data indicate the involvement of αCA4 in the photosynthetic reactions in the thylakoid membrane, in particular in processes associated with the protection of higher plants’ photosynthetic apparatus from photoinhibition.

## 1. Introduction

Carbon is the main element of most biomolecules, and its circulation in biochemical reactions is a necessary condition for maintaining life. Perhaps this is why carbonic anhydrases (CAs), the enzymes which accelerate the reaction of interconversion of CO_2_ and bicarbonate, CO_2_ + H_2_O ↔ H_2_CO_3_ ↔ HCO_3_^−^ + H^+^, with the release and consumption of a proton, are the ubiquitous enzymes, found in all living organisms, in almost all tissues and organelles. Extensive studies have revealed the important role of CAs in animal and human organisms and the impact of their dysfunction on the development of many pathologies [1,2]. In recent years, attention to the role of CAs in plants, primarily in the processes of photosynthesis occurring in chloroplasts, has been increased [3,4]. Various assumptions about the role of CAs in the reactions of photosynthetic electron transport and fixation of inorganic carbon were made in the literature (see ref. in [5]), but very few of these hypotheses are considered to be proven. In Arabidopsis chloroplasts there are several CAs of β- and α-families situated in the plasma membrane, cytoplasm, mitochondria and chloroplasts (see ref. in [5]). There are data on the CAs and CA activity in thylakoids of higher plants [6,7,8,9,10,11]. One of them is αCA4, the protein encoded by the *At4g20990* gene, which was found after the proteomic analysis of the proteins from Arabidopsis thylakoid membranes [12]. Ignatova et al. [13] showed that this CA is situated in the granal thylakoid membranes and this is the evidence that αCA2 is also located in the thylakoid membranes [10].

In our previous studies, it has been discovered that the *At4g20990* (*αCA4*) gene knockout in T-DNA induced mutants of Arabidopsis led to a decrease in non-photochemical quenching of chlorophyll *a* fluorescence (nPQ) in leaves [6,7,10,14]. This parameter reflects several processes, which lead to a decrease of the solar energy input into reaction centers of photosystems, first in Photosystem II (PSII). Solar energy is required for the conversion of inorganic carbon into organic compounds in photosynthesizing organisms. When the input of light energy is in excess of the capability to utilize this energy in the photosynthetic electron transport chain and in the processes of CO_2_ fixation, this can cause damage to the photosynthetic apparatus or photoinhibition [15]. The latter process leads to large crop losses, and this explains the great interest in studies on the means protecting the photosynthetic apparatus from photoinhibition. They include a number of self-protection mechanisms, such as reactive oxygen species scavenging, chloroplasts movement into the “shade”, and accumulations of protective pigments. However, the development of nPQ plays a predominant role in protection from photoinhibition. First of all, there is the development of energy-dependent nPQ, i.e., the absorbed light energy dissipation into heat that is stimulated by an influx of protons into the thylakoid lumen during the illumination. A central role in the latter process is played by both the protein PsbS, which is located in PSII supercomplexes, and the enzyme violaxanthin deepoxidase (VDE). The contribution to energy-dependent nPQ makes both PsbS protonation and the activation of VDE, catalyzing the conversion of violaxanthin to zeaxanthin [16,17,18]. In the above-mentioned studies, it was shown that the decrease of nPQ in *αCA4* gene knockouts is the result of the energy-dependent nPQ decrease.

In the mutants carrying insertions in the region of the *αCA4* gene, there are a number of structural changes in the thylakoid membrane. Compared to the WT plants, the content of nPQ triggering enzymes, PsbS protein and STN7 kinase, as well as pigments of the violaxanthin cycle, significantly increased in the mutants [6]. These changes were especially pronounced in plants grown under increased light intensity. It is quite possible that these are the structural changes which allowed plants to adapt to the absence of this enzyme. In the WT plants, the content of transcripts of the *αCA4* gene also significantly increased after the acclimation of plants to high light [19]. These data indicated αCA4 importance under high illumination, when protection from photoinhibition was especially required.

The methods of molecular biology make it possible to purposefully modify certain genes of interest in order to increase our knowledge and improve some economically valuable traits of important agricultural crops. The development and improvement of these methods allow, firstly, for the identification of the mechanisms underlying the responses controlled by a large number of plant genes [20]. Mutations are a convenient tool for the identification of the function of the proteins encoded by the genes, in which normal functioning has been disrupted by this insertion [21]. There has been some progress in this direction due to the application of transfer-DNA (T-DNA) insertion mutants to evaluate the functions of plant CAs [11,22,23].

A wide range of mutations has been obtained by integrating exogenous DNA fragments into the *A. thaliana* genome using genetic transformations [24]. To date, these mutants are presented by three broad collections of T-DNA insertions (SALK, SAIL, and WISC). In plants with mutant phenotypes, a fragment of exogenous DNA acts as a marker that allows the cloning of DNA regions adjacent to the insertion. The genomes of these mutants were the subject of further investigation by the cloning of their large extended DNA regions adjacent to the insertion, followed by their sequencing with the identification of various disorders: translocations, inversions, deletions, and others [25,26,27]. In some cases, the insertions were accompanied by intra- and interchromosomal rearrangements, as well as by the inactivation of foreign DNA integration regions and changes in chromatin marks [26]. The phenotyping of the mutant line SALK_008491 created by T-DNA and known as nhd1-1, which should presumably have knocked out gene of plastid Na^+^/H^+^ antiporter, showed that in a fluctuating light regime its growth rate decreased and this was accompanied by PSII damage [28]. More intense study of the nucleotide sequence in the insertion region of the SALK_008491 line permits authors to reveal a 14 kb deletion affecting five genes upstream of the NHD1 locus on chromosome 3 (Chr3). Besides NHD1, the stromal NAD-dependent D-3-phosphoglycerate dehydrogenase 3 (PGDH3) locus was knocked out. Thus, just the loss of two plastid proteins, PGDH3 and NHD1, increased the sensitivity of the studied line to dynamic light stress. Thus, despite the perspective of the application of the insertional mutations, which are able to lead to changes in phenotypic traits, this approach possesses some limitations associated with multiple chromatin rearrangements. More reliable results could be obtained by introducing knockouts into the regions of the genes of interest using the CRISPR/Cas9 method. Taking into account the fact that the effects of *αCA4* gene knockout described above could be determined by the presence of insertions not only in the gene of interest, we have made efforts to continue the study proposed earlier of the important role of αCA4 in plants in protecting the photosynthetic apparatus from damage under excessive light, i.e., from photoinhibition. The availability of data on the nucleotide sequence of genes of Arabidopsis, including genes encoding CAs, allows researchers to use this approach to obtain knockouts, in the functional part of the gene of interest.

In the present study, CRISPR/Cas9 genomic editing of *αCA4* gene in *A. thaliana* plants followed by the creation of a homozygous line with knocked out gene encoding αCA4 has been performed. A comparative analysis of characteristics of this mutant with those of two mutant lines carrying T-DNA insertions in the same gene has shown the analogous features of the manifestation of both types of mutations in comparison with the WT. The results of this study confirmed the involvement of αCA4 in the functioning of the photosynthetic apparatus in Arabidopsis, in particular, in the processes associated with the protection of the photosynthetic apparatus of higher plants from photoinhibition.

## 2. Results

### 2.1. Analysis of Plants Carrying Site-Specific Mutations. Production of Homozygous Lines

**Figure 1:**
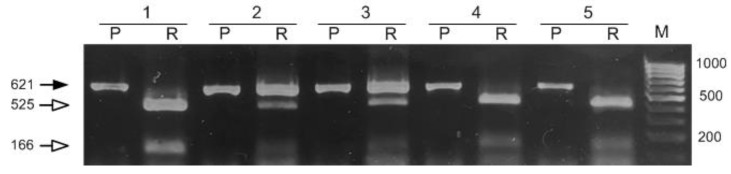
The electrophoretic pattern of separation of PCR fragments of the *αCA4* gene of T1 plants before and after treatment with an *Asp*S9I restriction enzyme in 1.5% agarose gel. Designations: 1–5 are the numbers of the corresponding plants; M-DNA marker Step 100 (Biolabmix, Russia), lengths of fragments in bp are indicated on the right; (P)—PCR fragment of the *αCA4* gene of the respective plants, and (R)—the result of *Asp*S9I restriction of the corresponding PCR fragment of the *αCA4* gene. The black arrow indicates the initial PCR fragment; the white arrows indicate the fragments obtained after *Asp*S9I digestion; the size of the fragments is indicated in bp.

According to the results of the electrophoretic separation of *αCA4* gene DNA fragments, isolated as described in Section 4, obtained by PCR with Ath *αCA4* Test1 F and Ath *αCA4* Test2 R primers and digested with *Asp*S91 restriction enzyme, two plants carrying the site-specific mutation in the heterozygous state were identified among twelve plants resistant to phosphinothricin. Figure 1 shows the electrophoregram of PCR fragments with DNA isolated from plants 1 to 5 before and after digestion with *Asp*S9I. The presence in lanes 2-R and 3-R of all three DNA fragments, the original (621 bp) and those digested by restriction enzyme *Asp*S9I (525 bp and 166 bp), indicates the heterozygous nature of the original DNA. This result shows that DNA in this sample was edited. In the case of plants 1, 4, and 5, there was complete digestion of the initial PCR fragment with *Asp*S9I restriction enzyme into two DNA fragments, the sizes of which were 525 bp and 166 bp. This indicated the absence of site-specific mutations in plants 1, 4, and 5.

**Figure 2:**
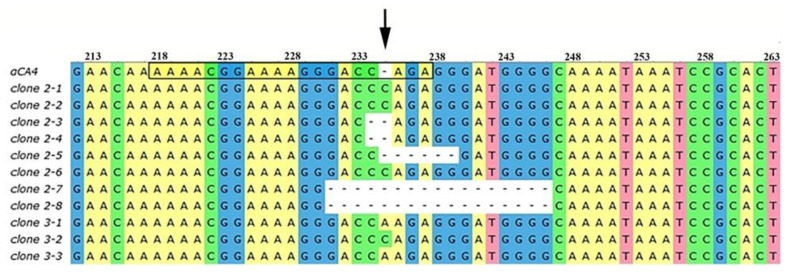
Alignment of the DNA sequences of the *αCA4* gene in the region of the putative mutation. Designations: *αCA4* is the sequence of the original gene; the numbers at the top are the numbers of the nucleotides in the gene; 20 bp *in the frame* is the *sequence of* the guide RNA*; Cas9* cleavage site is indicated by arrow; clones 2-1–2-8 are the clones of PCR fragments of the *αCA4* gene from plant 2; clones 3-1–3-3 are the clones of PCR fragments of the *αCA4* gene from plant 3.

Based on the results of the sequencing of individual clones of PCR fragments of the *αCA4* gene from plants 2 and 3, site-specific editing of the *αCA4* gene was confirmed. Figure 2 shows the results of the alignment of the DNA sequences of the *αCA4* gene in the region of the putative mutation obtained by the cloning of the PCR fragments of the *αCA4* gene from plants 2 and 3 with the sequence of the original gene. Among the clones sequenced, we have found six clones carrying an insertion of one nucleotide, nucleotide C in four clones (2-1, 2-2, 2-6 and 3-2) and nucleotide A in two clones (3-1 and 3-3), two clones carrying a deletion of one nucleotide C (2-3 and 2-4), one clone carrying a deletion of five nucleotides (2-5) and two clones carrying a deletion of seventeen nucleotides (2-7 and 2-8). All identified mutations are deletions or insertions leading to a shift in the reading frame of the original gene and, accordingly, to its knockout (Figure 2).

**Figure 3:**
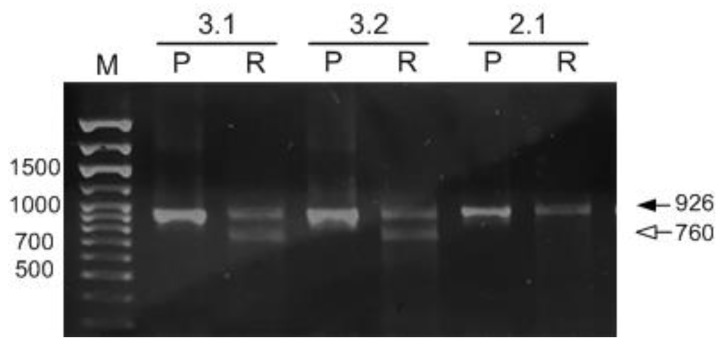
The electrophoretic pattern of separation in 1.5% agarose gel of PCR fragments of the *αCA4* gene of T2 plants before and after treatment with *Asp*S9I restriction enzyme. Designations: 3.1, 3.2, 2.1 are the numbers of the respective plants; M-DNA marker Step 100 Long (Biolabmix, Russia), lengths of fragments in bp are indicated on the left; (P)—PCR fragment of the *αCA4* gene of the respective plants, and (R)—*Asp*S9I restriction of the corresponding PCR fragments of the *αCA4* gene. The black arrow indicates the initial PCR fragment; the white arrow indicates the fragment obtained after *Asp*S9I digestion. Sizes of the fragments are indicated in bp.

To obtain homozygous lines carrying a mutation in the *αCA4* gene, the offspring from self-pollination of plants 2 and 3 were planted in the soil and analyzed individually with primers Ath *αCA4* Test1 F and Ath *αCA4* Test1 R (See Section 4). Among the analyzed 16 plants, one homozygous plant was identified. It corresponds to plant number 2.1. A PCR fragment of the *αCA4* gene, which was obtained using DNA isolated from plant 2.1, after digestion by *Asp*S9I restriction enzyme, remained unchanged (Figure 3). This result indicated the presence of site-specific mutation in both copies of the target gene. Seeds collected from plant 2.1 (Crispr_α4) were used for further study.

### 2.2. Photosynthetic Characteristics of Arabidopsis Plants Lacking αCA4

**Figure 4:**
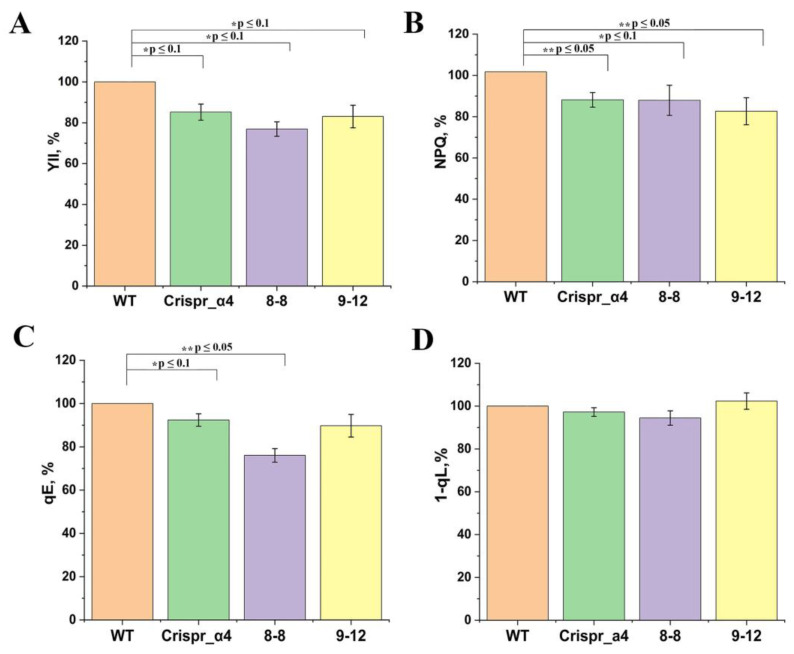
The effect of αCA4 knockout on the quantum yield of PSII, Y_II_ (**A**), total non-photochemical quenching of chlorophyll a fluorescence, NPQ (**B**), the coefficient of energy dependent component of nPQ, qE (**C**), and the coefficient 1-qL (**D**) in intact leaves of the WT Arabidopsis plants and αCA4-KO: Crispr_α4, 8-8 and 9-12. The calculations of the parameters are presented in Section 4. The values were measured in 7 min after illumination with continuous light at a photon flux density of 530 μmol quanta/(m^2^ s) PAR and at ambient air CO_2_ concentration. The 100% values were the data for the WT plants: Y_II_ of 0.13, NPQ of 1.59, qE of 0.29 and 1-qL of 0.66. Significant differences are denoted as * *p* ≤ 0.1, ** *p* ≤ 0.05.

The changes in plants lacking αCA4, which were obtained using Crispr/Cas9 method, were compared with those in αCA4-KO obtained using T-DNA insertion vs. WT plants. We have found somewhat decreased values of PSII quantum yield (Y_II_) measured under conditions of high light intensity and ambient CO_2_ concentration in all three αCA4-KO lines (Figure 4A). The total level of nPQ of chlorophyll *a* fluorescence expressed as NPQ measured under conditions of high light intensity in all three αCA4-KO lines was 10–15% lower than in the WT plants (Figure 4B), and the energy dependent component of nPQ, qE, was 10–30% lower in mutants of all three lines vs. WT plants (Figure 4C). The energy dependent component of nPQ, expressed as coefficient qE, which had been measured as the part nPQ relaxation in 1 min after light was switched off (see Material and Methods) and associated with relaxation of the PsbS protein conformational changes, was 10–30% lower in mutants of all three lines vs. WT plants (Figure 4C). The knockout of *αCA4* gene had no effect on both NPQ and qE when measured at low light intensity (not shown). Thus, the previous findings in the experiments with the insertion of Arabidopsis mutants with suppressed αCA4 synthesis on the effect of the nPQ reduction [7,14] were also observed in Crispr_α4 mutants. αCA4-KO mutants of all three lines, as compared with WT plants, had no differences in 1-qL parameter, which is the characteristic of plastoquinone pool redox state [29] (Figure 4D).

### 2.3. Daily Dynamics in Starch Content in αCA4-KO

The starch content, measured immediately after turning on the light, was 15–35% lower in αCA4-KO of all three mutant lines than in WT (Table 1). After 8 h of illumination in WT leaves it became twice as high, while in the mutants of all three lines the starch content increased 4.6–4.9 times compared with that in these plants immediately after the start of illumination (Table 1). The higher increase in the starch content in αCA4-KO leaves than in WT leaves is in agreement with the previously obtained data [14]. Taking into account the levels of starch content in the leaves after the dark period, night, it may be concluded that both the intensity of synthesis during the light period, as well as the starch utilization during the dark period, were significantly accelerated in αCA4-KO vs. WT plants.

### 2.4. Effect of αCA4 Absence on the Expression Levels of the Cytoplasmic and Chloroplast Carbonic Anhydrases

We have studied the effect of the *αCA4* gene knockout on the expression level of several genes encoding CAs located in the cytoplasm, chloroplast stroma, and thylakoid membrane. From four alternative splicing forms of *βCA1* gene, there are two pairs with the same sequences on 3′ ends: *βCA1.1* and *βCA1.2*, and *βCA1.3* and *βCA1.4* [30]. Therefore, two forms of *βCA1.1* and *βCA1.2*, encoding the stromal βCA1, were determined together (*βCA1.1 + 1.2*), as well as the other two, βCA1.3 and βCA1.4 (*βCA1.3 + 1.4*). Since the GFP fluorescence signal for βCA1.3, located in the chloroplast envelope, was much lower than that of βCA1.4, located in the cytoplasm [30], we consider *βCA1.3+1.4* as transcripts encoding the cytoplasmic form of βCA1.

**Figure 5:**
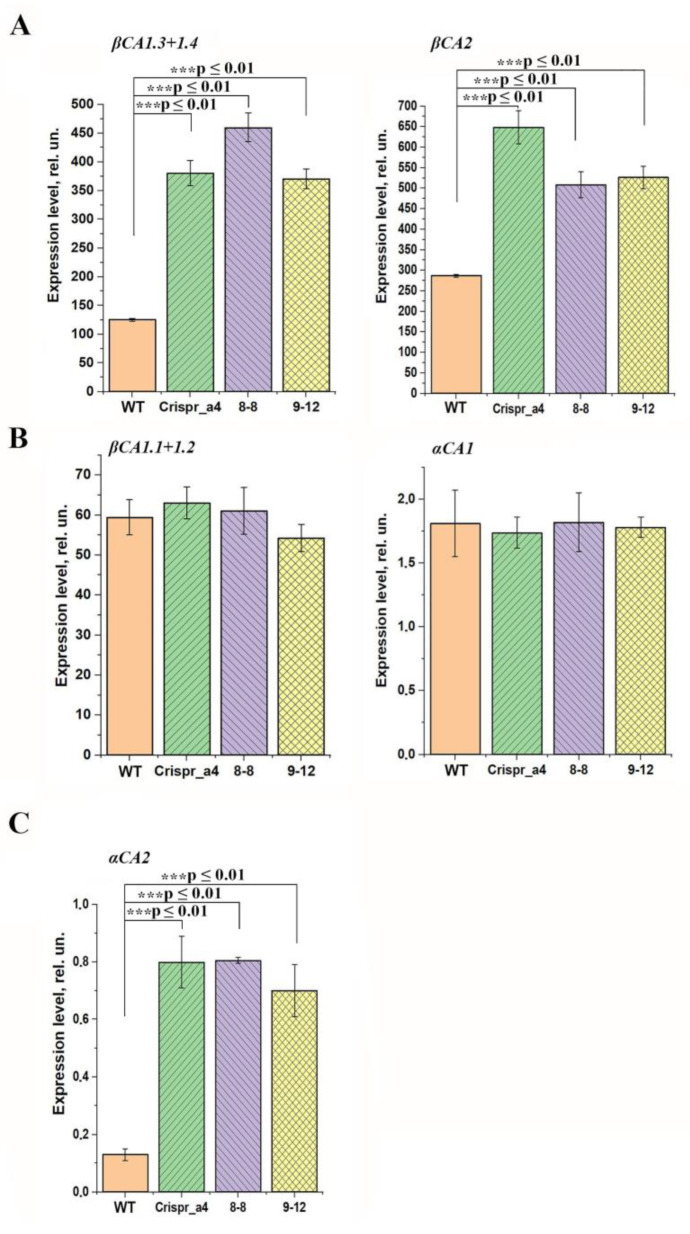
The expression level of the genes encoding cytoplasmic CAs (**A**): *βCA1.3 + βCA1.4* and *βCA2*; stromal CAs; (**B**): *βCA1.1 + βCA1.2* and *αCA1*; (**C**) thylakoid *αCA2* in Arabidopsis WT plants and αCA4-KO lines: Crispr_α4, 8-8 and 9-12. Data were normalized for actin gene expression. Values are means ± S.E (n = 3) of three independent experiments. Significant differences were denoted as *** *p* ≤ 0.01.

The knockout of αCA4 encoding gene has led to about a 2.5–3 times increase in the content of transcripts of CA genes encoding cytoplasmic ones: *βCA1.3 + 1.4* and *βCA2* in all three lines of mutants (Figure 5A). The expression levels of the genes encoding the stromal CAs, βCA1.1 + 1.2, as well as of one more chloroplast CA αCA1, in all three lines of αCA4-KO, were the same as in the WT plants (Figure 5B). In these mutants, the level of expression of the gene encoding the thylakoid αCA2 has been significantly, 5.5-6-fold, increased (Figure 5C).

### 2.5. Influence of the Knockout of αCA4 Gene on the Changes in the Content of Lhcb Encoding Genes Transcripts

**Figure 6:**
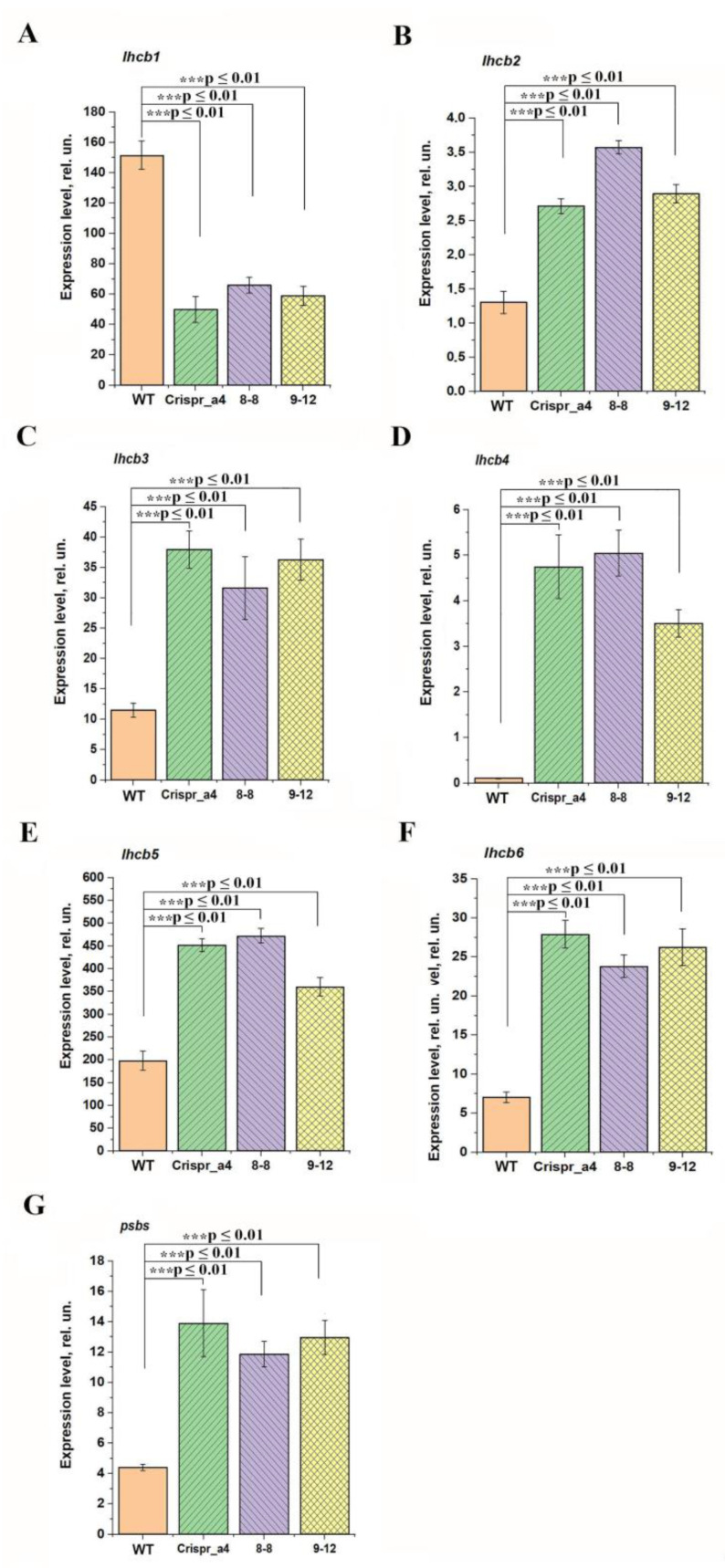
The expression level of *lhcb1* (**A**), *lhcb2.2* (**B**), *lhcb3* (**C**), *lhcb4* (**D**), *lhcb5* (**E**), *lhcb6* (**F**) and *psbs* (**G**) genes in leaves of Arabidopsis WT plants and αCA4-KO lines: Crispr_α4, 8-8 and 9-12. Data were normalized for actin gene expression. Data are shown as mean ± the SE (n = 3). The experiments were performed three times with similar results. Significant differences were denoted as *** *p* ≤ 0.01.

Previously, we have found that the content of the proteins Lhcb1 and Lhcb2 of PSII light harvesting complex (LHCII), as well as the expression level of the genes encoding these proteins, was changed in αCA4-KO if compared with WT plants [7]. In the present study, we show that the expression levels of the genes encoding the proteins of PSII light harvesting antenna, *lhcb1*, *lhcb2*, *lhcb3*, *lhcb4*, *lhcb5*, *lhcb6* and *psbs*, were different in αCA4-KO of all three lines if compared with WT. *lhcb1* gene expression was three times lower (Figure 6A) and the expression levels of all other genes, encoding proteins of light harvesting antenna, Lhcb2, Lhcb3, Lhcb4, Lhcb5 and Lhcb6, were more than two times higher in all three mutant lines vs. WT (Figure 6B–F). The excess of the level of expression of *lhch4* gene in all three αCA4-KO lines was significantly, by two orders of magnitude, higher if compared with the WT (Figure 6D). The expression level of the gene encoding the PsbS protein, which is the main protein inducing the rearrangements in LHC, leading to the development of nPQ, was also higher in αCA4-KO than in the WT (Figure 6G). This correlates with our earlier data on an increase in the content of PsbS in αCA4-KO protein [6].

Thus, the knockout of the gene encoding αCA4 obtained using Crispr/Cas9 method showed changes similar to those observed in T-DNA insertion mutants grown under the same conditions. It is possible that changes in the content of the proteins of PSII antenna are associated with adaptive rearrangements in mutants associated with a decrease in nPQ in the absence of αCA4.

**Figure 7:**
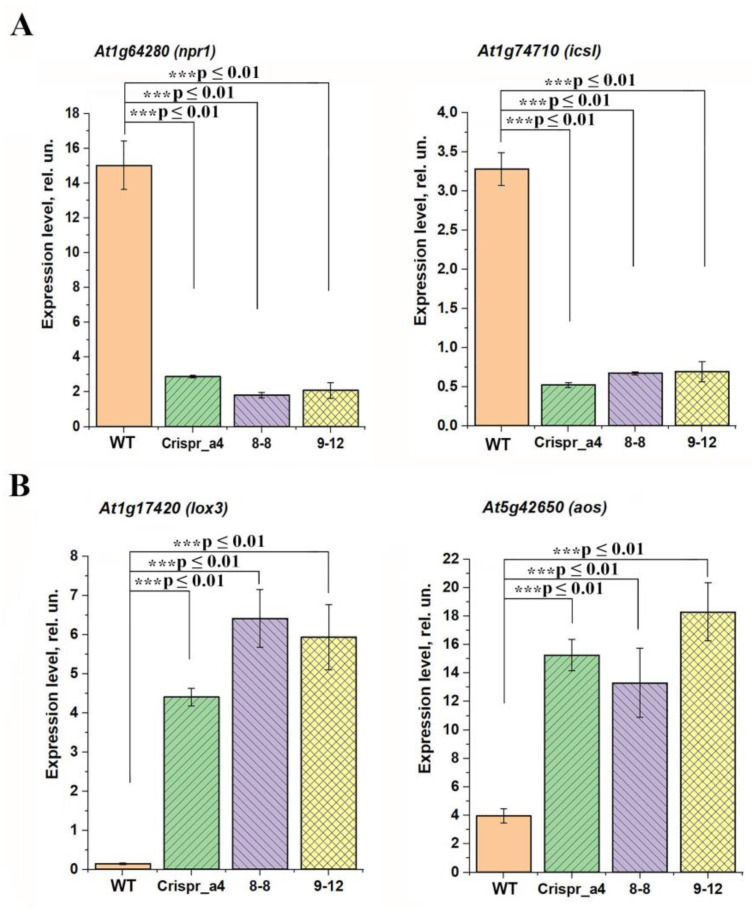
The content of transcripts of the genes inducible by plant immune signals in Arabidopsis WT plants and αCA4-KO lines: Crispr_α4, 8-8 and 9-12. (**A**) *At1g64280* (*npr1*) and *At1g74710* (*icsI*), induced by salicylic acid; (**B**) *At1g17420* (*lox3*) and *At5g42650* (*aos*), induced by jasmonic acid. Data were normalized for actin gene expression. Values are means ± S.E. (n = 3) of three independent experiments. Significant differences were denoted as *** *p* ≤ 0.01.

It is possible that a signal for a change in the intensity of expression of the genes encoding the proteins of PSII LHC are the changes in the level of the molecules involved in intracellular and intercellular signaling. The expression of the genes, which are the markers of induction of stress transcriptional cascades activated by the molecules of phytohormones salicylic acid (SA) and jasmonic acid (JA), were also different in αCA4-KO vs. WT (Figure 7). The expression levels of the genes induced by SA, *At1g64280* (*npr1*) and *At1g74710* (*icsI*), were lower in αCA4-KO (Figure 7A), whereas the expression level of JA-induced genes, *At1g17420* (*lox3*) and *At5g42650* (*aos*), were higher in these mutants than in the WT (Figure 7B). These data indicate that the hormonal status of mutants lacking αCA4 differs from that of WT. The JA-induced systemic acquired resistance (SAR) pathway is activated, while the SA-mediated SAR pathway, which is an antagonist of JA [31], is suppressed.

## 3. Discussion

The genetic construct used in our work was especially designed for editing the Arabidopsis genome using the floral dip method [32]. The Cas9 endonuclease gene was under the control of the RPS5a promoter, which provided a high level of gene expression in germline cells at the stage of agrobacterial transformation of *A. thaliana* plants [33]. The frequency of mutations in the *αCA4* gene with selected guide RNA (gRNA) was of 16.6% (two plants from twelve), which allowed us to successfully identify plants carrying the edited gene (Figure 1). The successful choice of gRNA also obviously contributed to the high editing efficiency. According to the CRISPOR resource, the chosen gRNA was characterized by a high probability of the occurrence of mutations leading to a shift in the reading frame. This probability was calculated by the Lindel repair model [34] and is determined by the context of the surrounding of the site of the putative mutation. Sequencing of the clones of PCR fragments of *αCA4* gene isolated from the edited plants showed that all identified gene mutations are deletions or insertions leading to a reading frame shift (Figure 2). Typically, such kinds of mutations either arrest the synthesis of a protein chain or yield defective protein molecules without a decrease in the transcription of the mutant gene.

When using CRISPR/Cas9, different copies of a targeted gene may be mutated at different times. Depending on the Cas9 delivery system, this can occur at different stages and provides opportunities and challenges for isolating plants [35]. Arabidopsis T1 mutants display an extremely low frequency of homozygous mutations in the first generation, even with germline-specific Cas9 promoters, with homozygous mutants recovering with an increasing frequency in later generations [36,37]. In our case, plants homozygous for the *αCA4* gene mutation were identified in the T2 generation (Figure 3), which also reflects the high efficiency of the tools used.

The comparison of the characteristics of these mutant plants with knocked out gene encoding αCA4 obtained using CRISPR/Cas9 genome editing system, as well as the mutants obtained using the method of T-DNA insertions, showed their similarity (Figure 4, Figure 5, Figure 6 and Figure 7, Table 1). The NPQ in all kinds of mutants was lower (Figure 4B) than in the WT due to a decrease in the energy-dependent quenching component qE (Figure 4C), which depends on the degree of protonation of the PsbS protein [16,17]. This difference was manifested in conditions of increased illumination, that is, when protection from photoinhibition is especially necessary. We have previously shown that it was high illumination that led to an increase in the expression level of the gene encoding aCA4 [19]. This difference was manifested in conditions of increased illumination, that is, when protection from photoinhibition was especially necessary. It is important that the parameter 1-qL, which indicates the redox state of the PQ-pool and can serve as an indirect indicator of the concentration of protons in the lumen during illumination, and which determines the rate of PQH_2_ oxidation, was the same as in WT in all three kinds of the mutants studied (Figure 4D). This was evidenced when, in αCA4-KO, the decrease in nPQ was not determined by a decrease in the proton concentration in the lumen, but rather by a decrease in the local protonation of the PsbS protein in the absence of αCA4. This is in accordance with our previous hypothesis about aCA4 operation in the membrane (see Figure 3 in [7]).

Being located in granal thylakoids close to PSII [13] accelerates the hydration of freely penetrating CO_2_ to the hydrophobic membrane regions. The protons released in this reaction protonate the glutamate of PsbS protein; the latter process induces conformational changes in LHCII and leads to an increase in thermal energy dissipation. It has now been revealed that the knockout of the *At4g20990* gene was accompanied by an increase in the content of the transcripts of the gene encoding PsbS protein (Figure 6G) that corresponds with a significant, two-fold increase in the content of these proteins [6].

Previously, it was found that the contents of Lhcb1 and Lhcb2 proteins decreased by an average of 20% [2]. The decrease of the content of these proteins may be considered as the means to reduce the light energy input to reaction center under a lower level of nPQ. In the present study, we have found that in all three αCA4-KO lines the expression level of *lhcb1* gene was 60-70% lower than in WT, whereas the levels of expressions of genes encoding all other Lhcb encoding genes was higher in α-CA4-KO than in the WT (Figure 6). The difference in the expression level of the gene encoding minor protein of light-harvesting antenna PSII, Lhcb4, in all three αCA4-KO lines was particularly significant, two orders of magnitude higher than in WT (Figure 6D). These data are important since Lhcb4 is connected with PsbS in the PSII antenna, and their interaction is considered as a pH-dependent trigger of nPQ development [38,39].

In [2] the expression level of the *lhcb1* gene was not measured, and the results of the present study (Figure 6) show that, unlike other *lhcb* genes, this level decreases in mutants, which corresponds with the data in [2] on a decrease in the content of this protein. The results on the expression of the *lhcb2.2* gene were in full accordance with the results in [6] for plants grown under the same conditions as in the present work. In that study, attention was drawn to the fact that the amount of the corresponding protein in mutant plants was about 25% lower than in the WT in spite of an increase in the expression of the *lhcb2.2* gene. These data suggested that the regulation of this protein content is carried out not only at the transcriptional level, but also at the translational level. It was established that the regulation of the antenna protein synthesis can occur at both the translational level [40] and the transcriptional level [41]. The difference in the control of the content of Lhcb1 and Lhcb2 proteins included in the LHCII trimers may be of physiological significance, for example, for the adjustment of adaptive antennae rearrangements.

In the present study, it was discovered that the level of transcripts of the genes encoding carbonic anhydrases βCA2, βCA1.3, βCA1.4 and αCA2 in αCA4-KO increased (Figure 5A,B,E). The increase in the expression level of *αCA2* gene was especially high; it was 5.5–6 times higher in αCA4-KO than on the WT (Figure 5C). For αCA2 there is evidence of its position in thylakoid membranes [10] as well as of αCA4 [12,13,42]. The significant increase in the expression of the *αCA2* gene in the absence of the latter can reflect the interrelation of the operation of these two CAs, as was proposed earlier [5]. The changes in the expression levels of cytoplasmic CAs and the absence of the changes in the expression levels of stromal CAs in αCA4-KO is the interesting challenge, which requires future study.

The knockout of the gene encoding αCA4 led to a gain in the amount of starch in the light relative to the dark level that was almost the same in all mutants, namely, 4.6–4.9 times compared to WT, where this increase was only 2.3 times (Table 1). The degradation of starch in the dark was also equally accelerated. The exact cause of these changes under the influence of mutation is not yet clear. An increase in starch degradation can reflect its increased output from the chloroplast into the cytoplasm that, in its turn, can lead to an increase in its use for the biosynthesis of cellular structures. A reflection of the latter process may be the increase in the size of T-DNA induced mutants found in [14]. Comprehensive analysis of starch metabolism in Arabidopsis [43] indicates the connection of biosynthesis and starch degradation systems. Bearing in mind the co-operative functioning of CAs located in plant cells [5], one can assume that the absence of aCA4 activity affected the functioning of other CAs of the chloroplast, in particular CAs involved in the transportation of compounds through the envelope into the cytoplasm. The role of CAs in this transportation has been identified in a number of studies [44,45].

In our experiments, we have also found that the expression level of the gene encoding the main NPR protein, NPR1, was lower in αCA4-KO than in the WT (Figure 7A), as well as in one more SA-induced gene, *At1g74710* [46]. At the same time, an increase in the expression intensity of the genes of the SA-antagonistic JA-mediated SAR pathway [31] was observed in αCA4-KO if compared with WT (Figure 7B). The ability of CAs of the β-family to interact directly with NPR transcription factors, which are required for the response to increase of the level of SA, was shown by Medina-Puche et al. [47]. The changes in the intensity of βCA synthesis, which are manifested in a change in the expression of their genes (Figure 5A), could lead to modulation in the perception of SA levels in mutant plants. The data of changes in SAR pathways in the absence of αCA4 (Figure 7) may provide the alternative explanation of observed changes in starch biosynthesis and degradation. There are data about the effects of plant hormones on the expression of genes involved in starch metabolism in potatoes [48].

Despite the perspective of application of the insertional mutations, which are able to lead to changes in phenotypic traits, nevertheless, this approach possesses some limitations associated with multiple genome rearrangements. The results presented in the present study are the first example of successful knockout in *A. thaliana* of the CA encoding gene, using the CRISPR/Cas9 genome editing method that is permitted to check and validate the role of αCA4 as the important participant in the photosynthetic process in the higher plant. Overall, the appropriate choice of the methods and tools of Cas9 genome editing gives the opportunity to obtain site-specific mutations in a precise region of *A. thaliana* genome in a more promising method than T-DNA-induced mutagenesis. This study also opens up perspectives for the further application of this method for the knockout of genes encoding various CAs in plants.

It is known that CAs usually function in cooperation when providing a particular process. This can be observed in the example of mitochondrial CAs [49], and it seems that this is also the case in chloroplasts [5]. The potential outcome of using this approach to switch off genes encoding different types of CAs in *A. thaliana* is that the use of CRISPR/Cas9 editing in the multiplex variant [20] can lead to the knockout of two or more genes in the genome of one plant. Thus, researchers may have an excellent opportunity to produce mutant plant lines with multiple knockouts of chloroplast CAs genes, since these enzymes are convenient models for elucidating their functions and contributions to plant responses to different stresses. Obviously, this approach can be applied not only to CAs, but also to enzymes that function sequentially or in parallel in various metabolic pathways.

## 4. Materials and Methods

### 4.1. Plant Material and Growth Conditions

Experiments were performed with *A. thaliana* (L.) ecotype Columbia-0 (WT) and α-CA4 knockout plants (αCA4-KO) of three lines. The seeds of the mutants were obtained from the Arabidopsis Biological Resource Center (The Ohio State University Rightmire Hall 1060 Carmack Road Columbus, OH 43210, USA) as T-DNA insertion lines (SALK_117962 and SALK_024517C), and homozygous mutant plants containing the T-DNA insert in the *At4g20990* gene were used for further studies (“8-8” and “9-12” lines, respectively). The mutants were different only in the position of the gene knockout insertion. Seeds of *A. thaliana*, WT and mutants, were placed on soil. Seedlings of three week-old plants, WT, Crispr_α4, 8-8 and 9-12, were transplanted into pots, one per pot, containing a commercially available soil mixture. Plants were grown for 7–8 weeks in a chamber at 19 °C with a short day photoperiod (8 h day/16 h night) under a photosynthetically active radiance (PAR) of 80 μmol quanta m^−2^ s^−1^.

### 4.2. Plasmids for Gene Editing

Plasmids pDGE331 (Addgene No. 153240) for the intermediate stage of cloning and pDGE347 (Addgene No. 153228) with the Cas9 endonuclease gene under the control of RPS5a, an *A. thaliana* promoter, were received as a gift from J. Stuttmann [32]. The presence in the construct of the bar gene, which gives plant cells resistance to phosphinothricin, ensured the selection of T0 generation plants.

### 4.3. Selection of gRNA

Selection of the gRNA sequence for targeted modification of *At4g20990* (*αCA4*) gene was carried out using the resource CRISPOR.org [50]. After preliminary analysis, a guide sequence, located at the beginning of the second exon of the *αCA4* gene and starting at 218 bp of the gene, was chosen. Table 2 lists the oligonucleotide sequences used to assemble the gRNA (Ath guide218 F, Ath guide218 R).

### 4.4. Creation of the Genetic Construct pDGE347-218 to Introduce Double-Strand Breaks in the αCA4 GENE Sequence

Selected sequences that determine the specificity of the gRNA were transferred to the plasmid pDGE347 using the intermediate plasmid pDGE331. The second assembly step was to transfer the resulting gRNA carrying the guide sequence into the pDGE347 plasmid. Insertion into the plasmid pDGE347 was carried out at the restriction sites of the BsaI endonuclease (New England Biolabs, Ipswich, MA, USA). To confirm insertion of the target sequence, DNA of the resulting clones was sequenced using the pDGE forward primer (Table 2).

### 4.5. Transformation of A. thaliana

Agrobacterial transformation using the floral dip method (*Agrobacterium tumefaciens* strain GV3101) was performed according to the standard protocol [51] followed by seed selection on a standard Murashige–Skoog (MS) medium containing phosphinothricin (50 mg/L) as a selective agent.

### 4.6. Analysis of Site-Specific Mutations in the αCA4 Gene

For identification of site-specific mutations in the *αCA4* gene, 12 DNA samples isolated from individual T1 generation plants (Plants 1–12) resistant to phosphinothricin were analyzed. Genomic DNA was isolated using a CTAB buffer according to the Allen protocol [52]. Site-specific mutations were detected by restriction analysis DNA fragments of the *αCA4* gene obtained using PCR with Ath α*CA*4 Test1 F and Ath α*CA*4 Test1 R or Ath α*CA*4 Test2 R primers (Table 1). The gRNA sequence contains a restriction site for *Asp*S9I restriction enzyme (SibEnzyme, Novosibirsk) (GGNCC). Thus, in case of successful editing, the recognition site of this restriction enzyme is disturbed. That makes it possible to detect editing by the restriction of PCR fragment of the target gene. The size of the expected PCR fragment with primers Ath *αCA4* Test1 F and Ath *αCA4* Test1 R was 926 b.p. The primers were selected in such a way that digestion of the PCR fragment of the *αCA4* gene with the *Asp*S9I restriction enzyme led to the formation of two DNA fragments of 166 bp and 760 bp. The size of the expected fragment obtained using primers Ath *αCA4* Test1 F and Ath *αCA4* Test2 R would be 621 bp. Digestion by a restriction enzyme *Asp*S91 of the PCR fragment obtained with these primers has led to the formation of two DNA fragments of 166 bp and 525 bp. DNA samples, putatively containing the mutation, were sequenced after cloning of the target gene PCR fragments. Cloning of PCR fragments was performed using the InsTAclone PCR Cloning Kit (K1214, Fermentas), and each clone was sequenced individually. Sequencing was performed at the Evrogen company (Moscow, Russia).

### 4.7. Determination of Starch Content

Starch content was analyzed by measuring the absorbance at 620 nm of leaf aqueous extracts supplemented with KI after thorough washing of pigments [53]. The starch content was measured in the beginning of the light day and after 8 h of illumination.

### 4.8. Measurement of Chlorophyll a Fluorescence at Room Temperature

Parameters of chlorophyll *a* fluorescence were measured in unattached leaves, using a DUAL PAM-fluorometer (Walz, Germany) and calculated according to [54]. The plants were adapted to the dark for 2 h prior to measurement. Measurements were made at 530 μmol of quanta m^−2^ s^−1^ of actinic light from intrinsic source. The saturating light pulses (0.8 s, 8000 μmol quanta m^−2^ s^−1^) were applied in 7 min of illumination when the steady state level of fluorescence was achieved. The effective quantum yield of PSII, Y_II_, the parameter 1-qL, characterizing the redox state of the plastoquinone pool, and the parameter characterizing nPQ (NPQ) and the coefficient of energy-dependent nPQ, qE, were calculated as: Y_II_ = (F_m_′ − F_S_)/F_m_′, 1-qL = ((F_m_′ − F_s_)/( F_m_′ − F_0_′)) × F_0_′/F_s_, NPQ = (F_m_ − F_m_′)/F_m_′, and qE = (F_m1_ − F_m_′)/(F_m_ − F_0_), where F_m_ is the maximum fluorescence yield in response to a saturating pulse applied to dark-adapted leaves before illumination with actinic light, F_0_ is the minimal fluorescence level in dark-adapted state, F_0_′ is the minimal fluorescence level after the cessation of illumination, F_m_′ is the maximum fluorescence yield in response to a saturating pulse under illumination, F_s_ is the steady-state fluorescence yield, and F_m1_ is the fluorescence yield in response to a saturating pulse applied 1 min after the cessation of illumination.

### 4.9. Quantitative Reverse-Transcription PCR

Total RNA was extracted from frozen Arabidopsis leaves, using the Aurum total RNA Mini Kit (Bio-Rad, USA), and was treated with DNase to eliminate any genomic DNA contamination. cDNA synthesis was performed using a Reverse transcription kit OT-1 (Sintol, Russia) with oligo (dT) as a primer. The quantitative reverse-transcription polymerase chain reaction (qRT-PCR) was performed with qPCRmix-HS SYBR (Evrogen, Russia); the primer pairs specific for genes coding were Lhcb1 (forward 5′-AGAGGCCGAGGACTTGCTTTAC-3′ and reverse 3′-CCAAATGGTCAGCAAGGTTCTC-5′), Lhcb2 (forward 5′-ATGGCCACATCAGCTATCC-3′ and reverse 3′-CTCCAGTTAAGTAAGACGGTCTG-5′), Lhcb3 (forward 5′-AATGATCTTTGGTATGGACCTGAC-3′ and reverse 5′-CCACACGGACCCACTTTTG-3′), Lhcb4 (forward 5′- CAGCCGTACACTGAAGTCTTTGG -3′ and reverse 5′- TTCTATCCATATCAACGTCGTCAAC-3′), Lhcb5 (forward 5′- CGGATTGGATTTCGAGGACAAGCTACA-3′ and reverse 5′-GATAAAGAAACC GAGCATCGCAAACAT-3′), Lhcb6 (forward 5′-GCGATGGCAGCGGTTCTTG-3′ and reverse 3′-CCATGGCGTTGCCCACTC-5′), PsbS (forward 5′-CATTGGAGCTCTCGGAGACAGAGGAA-3′ and reverse 5′- CTCGTTCGCCTTCGTGAACCCAAACAAT-3′), LOX3 (*At1g74710*) (forward 5′-TCCAAGCGTGTGCTTACACCTC-3′ and reverse 5′-GTCCGTAACCAGTGATTGACAAG-3′), AOS (*At5g42650*) (forward 5′-GAGATTCGTCGGAGAAGAAGGAGAGAA-3′ and reverse 5′-AATCACAAACAACCTCGCCACCAAAA-3′), ICSI (*At1g74710*) (forward 5′-CAGCAGAAGAAGCAAGGCTT-3′ and reverse 5′-TCAATGCCCCAAGACCCTTTT-3′), NPR1 (*At1g64280*) (forward 5′-GGAGAAGACGACACTGCTGAGAAA-3′ and reverse 5′-CACCGACGACGATGAGAGAG-3′), *At5g09810* (actin 7) (forward 5′-GAAGGCTGGTTTTGCTGGTGAT -3′ and reverse 5′-CCATGTCATCCCAGTTACTTACAATACC-3′). Primers sequences for the genes encoding CAs: *At3g01500.1*+ *At3g01500.2* (*βCA1.1 + 1.2*), *At3g01500.3* + *At3g01500.4* (*βCA1.3 + 1.4*), *At5g14740* (*βCA2*), *At3g52720* (*αCA1*), *At3g52720* (*αCA2*) were used according to [19] with primers for *βCA1.1 + 1.2* corresponding to *βCA1a* primers, and primers for *βCA1.3+1.4* corresponding to *βCA1b* primers. The qRT-PCR data were normalized against housekeeping gene actin 7 gene. PCR reactions were run in LightCycler 96 Instrument, Roche Diagnostics GmbH (Switzerland).

### 4.10. Statistical Analyses

Statistical analyses were conducted using OriginPro. An ANOVA analysis with subsequent means comparison using the Holm–Bonferroni method was performed for Figure 4, Figure 5, Figure 6 and Figure 7. Significant differences were denoted as * *p* ≤ 0.1, ** *p* ≤ 0.05, *** *p* ≤ 0.01.

## 5. Conclusions

Using the CRISPR/Cas9 genome editing method, *A. thaliana* homozygous mutant line with knocked out *At4g20990* gene encoding αCA4 was obtained. By measuring the photosynthetic characteristics of these mutants, it was shown that the effects of this mutation coincide with the effects of the knockout of this gene in T-DNA insertion mutants. The data confirm the important role of αCA4 previously established on the basis of investigation of the latter. This role is the protection of photosynthetic apparatus from photoinhibition by participating in the development of energy-dependent dissipation of light energy, which manifests in the enhancement of the non-photochemical quenching of chlorophyll fluorescence.

## Figures and Tables

**Table 1:** The starch content in leaves of WT *Arabidopsis thaliana* and mutant plants with knocked out *At4g20990* gene encoding αCA4 (lines Crispr_α4, 8-8 and 9-12) measured at the beginning of the light day (after 16 h of darkness) and at the end of the light day (after 8 h of light). The 100% value is the starch content in the WT leaves at the beginning of the light day (1.17 mg/g of fresh leaves weight).

Plants		Starch Content, %	
		After 16 h of Darkness	After 8 h of Light
WT		100	232.0 ± 25.6
αCA4-KO	Crispr_α4	63.2 ± 5.9	295.7 ± 38.5
	8-8	80.1 ± 11.4	390.1 ± 11.4
	9-12	85.6 ± 14.9	392.0 ± 15.4

**Table 2:** Oligonucleotides used for gRNA assembly.

Name of the Oligonucleotide	Nucleotide Sequence 5′-3′
Ath guide218 F	ATTGAAAACGGAAAAGGGACCAGA
Ath guide218 R	AAACTCTGGTCCCTTTTCCGTTTT
pDGE forward	CGAATCAAAAGTTGAGCTCC
Ath *αCA4* Test1 F	ATTTCACACGCTCATTCTGAAGTCG
Ath *αCA4* Test1 R	AAGGAGGAACAGTGAGAGAGCCAA
Ath *αCA4* Test2 R	GGTCGTACCTGTAATAATGCCAAAT

## Data Availability

Not applicable.
